# Pilot Investigation of the Circadian Plasma Melatonin Rhythm across the Menstrual Cycle in a Small Group of Women with Premenstrual Dysphoric Disorder

**DOI:** 10.1371/journal.pone.0051929

**Published:** 2012-12-19

**Authors:** Ari Shechter, Paul Lespérance, N. M. K. Ng Ying Kin, Diane B. Boivin

**Affiliations:** 1 Centre for Study and Treatment of Circadian Rhythms, Douglas Mental Health University Institute, Department of Psychiatry, McGill University, Montréal, Québec, Canada; 2 Department of Psychiatry, Centre Hospitalier de l’Université de Montréal, Université de Montréal, Montréal, Québec, Canada; 3 Clinical Research Division, Douglas Mental Health University Institute, Department of Psychiatry, McGill University, Montréal, Québec, Canada; University of Western Brittany, France

## Abstract

Women with premenstrual dysphoric disorder (PMDD) experience mood deterioration and altered circadian rhythms during the luteal phase (LP) of their menstrual cycles. Disturbed circadian rhythms may be involved in the development of clinical mood states, though this relationship is not fully characterized in PMDD. We therefore conducted an extensive chronobiological characterization of the melatonin rhythm in a small group of PMDD women and female controls. In this pilot study, participants included five women with PMDD and five age-matched controls with no evidence of menstrual-related mood disorders. Participants underwent two 24-hour laboratory visits, during the follicular phase (FP) and LP of the menstrual cycle, consisting of intensive physiological monitoring under “unmasked”, time-isolation conditions. Measures included visual analogue scale for mood, ovarian hormones, and 24-hour plasma melatonin. Mood significantly (P≤.03) worsened during LP in PMDD compared to FP and controls. Progesterone was significantly (P = .025) increased during LP compared to FP, with no between-group differences. Compared to controls, PMDD women had significantly (P<.05) decreased melatonin at circadian phases spanning the biological night during both menstrual phases and reduced amplitude of its circadian rhythm during LP. PMDD women also had reduced area under the curve of melatonin during LP compared to FP. PMDD women showed affected circadian melatonin rhythms, with reduced nocturnal secretion and amplitude during the symptomatic phase compared to controls. Despite our small sample size, these pilot findings support a role for disturbed circadian rhythms in affective disorders. Possible associations with disrupted serotonergic transmission are proposed.

## Introduction

Mood is influenced by a non-additive interaction between circadian phase and duration of awakening [1]. A chronobiological basis of affective disorders was proposed, and dysregulated circadian rhythms in psychiatric disorders, including major depressive disorder (MDD), seasonal affective disorder (SAD), bipolar disorder, and schizophrenia were observed [2], [3], [4]. Clock gene abnormalities are also implicated in bipolar disorder and schizophrenia [5], [6].

We observed [7] that an interaction between menstrual and circadian processes alters the circadian variation of rapid eye movement (REM) sleep and core body temperature (CBT) during the luteal phase (LP) compared to the follicular phase (FP). Furthermore, women are up to twice as likely as men to suffer from affective disorders [8]. This raises the question of whether a circadian and menstrual interaction also influences mood in women, and whether altered circadian rhythms contribute to the development of premenstrual dysphoric disorder (PMDD), a menstrual cycle-related mood disorder.

PMDD is a mood disorder affecting 3–8% of women [9]. The occurrence of PMDD is cyclical and defined by ovarian hormone status across the menstrual cycle, with symptoms occurring during LP and remission after menses [10]. Among several symptoms, including sleep complaints, PMDD is marked by depression, tension, affective lability, and irritability of sufficient intensity to interfere with daily activities and relationships [10]. PMDD women can experience circadian rhythm changes [11], and a chronobiological basis for this disorder has been proposed [12].

In PMDD, it was hypothesized that ovarian hormone variations may alter the expression of the endogenous circadian pacemaker and precipitate mood disruption in predisposed females [12]. While chronobiological investigations in PMDD have been limited and inconsistent, authors noted abnormal melatonin rhythm in PMDD, including phase-advanced offset [13], shorter duration of secretion [13], and decreased levels [13], [14] compared to controls. Similar to MDD and SAD, PMDD women have responded to non-pharmacologic chronotherapies [11]. Light therapy during LP significantly reduced depressive symptoms in PMDD women [15], [16], [17], with a response rate of up to 60% for either morning or evening bright light [17] and in 89% of menstrual cycles treated with evening bright light exposure [15]. PMDD women also respond to both total and partial sleep deprivation with improvements in mood [18], [19], as was observed in 80% of women with premenstrual depression after total sleep deprivation [18] and up to 67% of patients after early-night partial sleep deprivation [19].

The importance of circadian rhythms and the sleep-wake cycle in the pathophysiology and potential treatment of PMDD therefore becomes apparent. Few studies have focused on circadian rhythms in PMDD. Here, we utilized a highly controlled experimental procedure in time-isolation designed to minimize confounding influences like postural changes, light levels, and meal intake on the observed melatonin circadian rhythms. We conducted a chronobiological characterization of PMDD, focusing on melatonin, one of the most widely used measures of the circadian pacemaker. Since PMDD symptoms appear during LP, we expected to see an altered circadian melatonin rhythm specifically during this menstrual phase.

To our knowledge, this pilot study is the first investigation of the endogenous circadian rhythm of melatonin in PMDD women under highly controlled time-isolation conditions. This line of investigation has clinical potential by clarifying the role of the endogenous circadian system in PMDD, and can lead to the further development of innovative chronotherapeutic options.

## Methods

### Participants

Five PMDD women (age range: 28–41 years old) and 5 control women (age range: 21–43 years old) were included in the study. PMDD diagnoses were based on the Structured Clinical Interview for DSM-IV (SCID), the Prospective Record of the Impact and Severity of Menstrual Symptoms (PRISM) [20] and a Visual Analogue Scale (VAS) [21] completed daily for ≥2 consecutive menstrual cycles. The reliability and validity of the VAS and PRISM as effective tools for the prospective tracking of menstrual cycle-related mood disturbance has been documented [20]. The 11-item VAS (100-mm bipolar scale, with 0 mm being “not at all” and 100 mm being “extreme symptoms”) was based on DSM-IV criteria for PMDD diagnoses [10], including the measures depressed mood, tension, affective lability, irritability, decreased interest, difficulty concentrating, lack of energy, change in appetite, change in sleep patterns, feeling out of control, and physical symptoms. An individual mean score for each of the four core PMDD symptoms (depressed mood, tension, affective lability, irritability) was calculated for days 6–10 after menstruation (FP) and also for the last five days of the menstrual cycle (late-LP). Eligibility criteria required the presence of at least five of the eleven overall symptoms during late-LP, and an increase of ≥200% on one, or ≥100% on two or more of the core symptoms for the mean late-LP score compared to FP. These diagnostic criteria were developed as prospective criteria to be met, which demonstrate an unambiguous and clinically relevant worsening of symptoms during late-LP compared to FP, with the VAS scores serving to illustrate prospective day-to-day objective differences in the DSM-IV diagnostic symptoms. The criteria are modeled after, but are more stringent than those used by Steinberg et al. [22] and Steiner et al. [21], which were a 50% worsening in three core symptoms or a 100% worsening of one core symptom. All potential PMDD participants met with a psychiatrist (P.L.) twice, at FP and late-LP, to confirm the diagnosis. All PMDD women included indicated insomnia symptoms selectively during the premenstrual phase with no clinical evidence of subjective sleep disruption during FP. Participants were excluded if diagnosed with another current Axis I disorder (i.e. clinical syndromes). From the PMDD group, Subject #2 reported one past episode of depression 3–4 years prior to study. SAD was ruled out by the Seasonal Pattern Assessment Questionnaire (SPAQ), a tool used for the assessment of seasonal variations in mood and behavior [23]. Axis II (i.e. personality) disorders were ruled out by clinical evaluation but not systematically with screening questionnaires. Age-matched controls completed the SCID and PRISM, and filled out the 11-item VAS for 2 consecutive menstrual cycles during screening and showed no evidence of PMDD or any other psychiatric disorder. Controls did not meet individually with the diagnosing psychiatrist, since none showed any evidence of PMDD based on VAS questionnaires or any other psychiatric disorder based on the SCID.

Participants were healthy and drug-free at the time of study. All had a history of regular menstrual cycles (range: 25–34±3 days), and ovulation was confirmed via plasma progesterone test scheduled on day 21 of the menstrual cycle preceding experimental procedures. All had no history of gynecological pathology, were ≥6 months post-partum, not currently breast feeding, and free of hormonal contraceptives. Participants had no history of night-shift work or transmeridian travel within three months of study. Before the experimental month, participants maintained a regular schedule of 8 hours sleep/darkness per day for at least three weeks, confirmed by a sleep-wake log, calls to the laboratory at bed/wake times, and wrist actigraphy for ≥1 week (Actiwatch, Mini-Mitter, Bend, OR). The Douglas Mental Health University Institute Research Ethics Board approved all procedures, which were in accordance with the Declaration of Helsinki, and all participants provided written informed consent.

### Design

Participants entered the laboratory twice for a 24-hour period of intensive physiological monitoring, comprising a constant posture (CP) procedure. Participants were studied individually in window-less, light- and temperature-controlled time-isolation suites. Throughout the CP, participants remained in constant conditions, including a maintained semi-recumbent posture, a time-cue free environment, iso-caloric snacks [24] served 1x/hour, and dim ambient light levels (<10 lux) throughout the waking period before and after the sleep episode. The CP included the requisite controls of a constant routine procedure [25] but allowed for a sleep episode since the current study was part of a larger investigation of nocturnal sleep across the menstrual cycle. The first visit was scheduled to occur within FP (mean days after menses onset ± SEM: 7.2±.59) and the second visit was scheduled to occur within LP (mean days after menses onset ± SEM: 21.4±.76). Participants entered the laboratory at ∼12∶00 to begin the CP, and were scheduled to have an 8-hour sleep episode based on their habitual bedtime. Nocturnal polysomnographic sleep was also recorded as part of a larger study on these control and PMDD participants, and has been reported elsewhere [26].

### Measures

Mood was measured during the screening phase with the 11-item VAS described above. Mean scores were calculated across days 6–10 after menstruation (FP) and across the last 5 days of the menstrual cycle (late-LP) for each of the four core PMDD symptoms individually (depressed mood, tension, affective lability, and irritability). The four core measures were averaged together to yield a mean score representing overall mood symptoms called the VAS-core [21], [22].

CBT was monitored (4x/minute) throughout the CP via a thermistor (Steri-Probe, Cincinatti Sub-Zero Products, Inc., Cincinnati, OH) inserted 10 cm into the rectum, connected to an in-house data acquisition system. Data were inspected visually and by an *ad hoc* program for probe malfunctions or “slips”, which were discarded.

Blood samples were collected 1x/hour via indwelling forearm catheter connected to an extension allowing frequent sampling without interrupting sleep. Between blood samples, heparinized-saline was infused (7.5 iu/cc at 30cc/hour) to reduce risk of clotting at the catheter insertion site. Heparinized-saline was cleared from the line before obtaining samples. A morning sample was assayed for estradiol and progesterone concentration. Assays were performed on the Beckman Coulter DxI 800 system, using Beckman reagents for chemiluminescence immunoassays (Beckman Coulter, Inc., Brea, CA; estradiol coefficient of variation [CV]: 10.7%; progesterone CV: 6.8%). Hourly samples collected throughout the CP were used to evaluate the circadian rhythm of plasma melatonin. Levels were determined by radioimmunoassay, using the LDN Melatonin Direct Assay Kit (Medicorp, Montreal, QC), a highly sensitive and specific assay which involves the use of ^125^I-labeled melatonin. The sensitivity of the assay is 2 pg/ml. The intra-assay CV is 9.9–12.3% for mean melatonin concentrations of 15–157 pg/ml, and the inter-assay CV is 9.6–16.2% for 21–205 pg/ml.

### Circadian Parameter Assessment

A dual-harmonic regression model [27] without serial correlated noise was applied to individual 24-hour CBT curves (1-min bins), and time of fitted CBT minimum was obtained from this model.

For melatonin, time of fitted maximum and amplitude were determined with a 3-harmonic regression model applied to individual melatonin curves [28]. Amplitude of the circadian curve was defined as the mean-to-trough difference of the first harmonic of the regression [29]. Melatonin circadian profile also included dim light melatonin onset (DLMOn), dim light melatonin offset (DLMOff), duration of secretion (time from DLMOn to DLMOff), and area under the curve (AUC; determined using trapezoidal method). DLMOn and DLMOff were defined as the clock-times of upward and downward crossing, respectively, of the threshold set at 25% of the fitted amplitude [30]. To generate 24-hour melatonin curves, each hourly data point throughout the CP was assigned a circadian phase from 0°−359.9°, relative to the fitted CBT minimum set at 0°. Data were collapsed per participant into 15° circadian bins (1 hour), and across participants per group and menstrual phase.

### Statistics

T-tests were used to compare participant characteristics including age, body mass index (BMI), and menstrual cycle length between groups. For VAS-core scores, ovarian hormones, and circadian parameters of melatonin (DLMOn, DLMOff, duration, time of fitted maximum, amplitude, and AUC), Kruskal-Wallis tests were used to determine main effects of group (Control vs. PMDD), Friedman’s tests were used to determine main effects of menstrual phase (FP vs. LP), and Kruskal-Wallis tests were used to determine the group x menstrual phase interaction. Significant interaction terms were further analyzed with Mann-Whitney tests for the group effect and Wilcoxon test for the menstrual phase effect. Three-way ANOVA for repeated measures (factors: group x circadian phase x menstrual phase) was used to analyze melatonin values across circadian phases. Simple main effects tests were used to analyze significant interactions.

In addition to these primary analyses, additional analyses were conducted in which the plasma melatonin results obtained from two healthy young women previously studied with a constant routine [31] and one healthy young woman studied with a CP [32], all during FP, were pooled with the 5 controls of the current study. This larger group of 8 controls was compared with the 5 PMDD women of the current study during FP. This was done in an attempt to increase sample size and further confirm results from our baseline group comparisons. No LP data were collected for the 3 supplementary control participants. As in the primary analyses, plasma melatonin data for these three women were assigned a circadian phase from 0°−359.9°, relative to their fitted CBT minimum set at 0°. Since blood was sampled less frequently in those studies (∼1x/2 hr), plasma melatonin data for these extra 3 women as well as the FP values for the 5 controls and 5 PMDD women of the current study were collapsed per participant into 30° circadian bins (2 hours), and across participants per group. Two-way between subjects ANOVA for repeated measures (factors: group x circadian phase) was used to analyze melatonin values across circadian phases during FP in PMDD women and the expanded group of controls. Simple main effects tests were used to analyze significant interactions. Unpaired-samples t-tests were used to analyze circadian parameters of melatonin (DLMOn, DLMOff, duration, time of fitted maximum, amplitude, and AUC) during FP in PMDD women and the expanded group of controls.

## Results

### Participant Characteristics

Controls (n = 5) and PMDD (n = 5) did not differ in age (t_8_ = -.71, P = .50), BMI (t_8_ = 1.62, P = .14), or menstrual cycle length (t_8_ = .00, P = .50) (Table 1). A significant group x menstrual phase interaction for VAS-core score was observed (H_1_ = 5.00, P = .025), with significantly increased late-LP values observed for PMDD compared to their own FP, and to controls (P≤.03, Wilcoxon and Mann-Whitney tests, respectively) (Table 1). Controls showed no menstrual phase variation for VAS-core scores (P = .41, Wilcoxon test). After the 3 supplemental controls were added to the original group of 5 controls (see *Statistics* in Methods section above) there were still no significant group differences in age (t_11_ = −1.58, P = .14) or BMI (t_11_ = .30, P = .77) (Table 2).

**Table 1:** Characteristics of controls and PMDD participants†.

Variable	Controls	PMDD women
**sample size**	5	5
**age, years**	30.4±3.67	33.6±2.68
**BMI, kg/cm^2^**	23.5±.58	22.02±.70
**menstrual cycle length, days**	26.4±1.03	26.4±.75
**VAS-core, mm**		
FP	3.12±1.73	2.11±.38
Late-LP	5.57±1.93	47.00±7.94 a
**progesterone, nmol/l**		
FP	9.53±1.17	7.46±.50
LP	44.47±11.30 b	51.73±10.32 b
**estradiol, pmol/l**		
FP	338.8±66.7	473.0±97.0
LP	719.8±193.3	486.6±141.1

PMDD: premenstrual dysphoric disorder; BMI: body mass index; FP: follicular phase; LP: luteal phase; VAS-core: mean score of visual analogue scale measures for depression, tension, affective lability and irritability values are mean ± SEM.

† Participant characteristics were also included in a related manuscript [26].

a indicates PMDD late-LP value is significantly (P≤.03) different than PMDD FP value and pone.0051929.g003.tifcontrol values in both menstrual phases.

b indicates LP values are significantly (P = .025) different than FP values.

**Table 2:** Characteristics of the expanded group of controls and PMDD participants†.

Variable	Controls	PMDD women
**sample size**	8	5
**age, years**	27.3±2.68	33.6±2.68
**BMI, kg/cm^2^**	22.3±.69	22.02±.70

† This table presents characteristics from the 5 control participants and 5 PMDD participants included in Table 1, but the control group is supplemented by demographics data from 3 other young healthy women who were studied under constant conditions in unrelated experiments. This larger group of 8 controls was compared to the 5 PMDD women of the current study during the FP in an attempt to increase sample size and further confirm results from our baseline group comparisons. Menstrual phase length, VAS-core scores, and progesterone and estradiol levels were not available for the 3 supplementary control participants. Values are mean ± SEM.

### Sex Hormones

No significant group x menstrual phase interaction (H_1_ = .175, P = .676) nor main effect of group (H_1_ = .011, P = .917) was seen for plasma progesterone. A significant main effect of menstrual phase was observed (χ^2^ = 5.00, P = .025), with progesterone values significantly increased during LP compared to FP in both groups (Table 1). No significant group x menstrual phase interaction (H_1_ = 3.153, P = .076), main effect of group (H_1_ = 0.011, P = .917), or main effect of menstrual phase (χ^2^ = .200, P = .655) was seen for plasma estradiol.

### Melatonin

#### Circadian profile

For AUC, a significant group x menstrual phase interaction was observed (Table 3) (H_1_ = 4.811, P = .028), with decreased values observed within PMDD women during LP compared to FP (P = .03, Wilcoxon test). For amplitude, no significant group x menstrual phase interaction, main effect of group, or main effect of menstrual phase was seen (Table 3). However, when compared only during LP, based on an priori prediction of reduced melatonin secretion during this phase, amplitude was reduced in PMDD compared to controls (P = .05, unpaired-samples t-test). No significant interaction, or main effects of group or menstrual phase, was observed for DLMOn, DLMOff, duration of secretion, and time of fitted maximum (Table 3), though a trend for a main effect of group was seen for duration of secretion (H_1_ = 3.153, P = .076).

**Table 3:** Circadian melatonin profile in controls and PMDD women.

	Controls, n = 5		PMDD women, n = 5					
	FP	LP	FP	LP		main effect of group	main effect of menstrual phase	group x menstrual phase interaction
**DLMOn, clock time**	20.18±.67	20.52±1.11	21.46±.66	21.66±.73	H_1_ = .525, P = .465		χ^2^ = 1.800 P = .180	H_1_ = .011, P = .917
**DLMOff, clock time**	9.10±.52	8.85±.16	8.29±.81	8.29±.80	H_1_ = 1.320, P = .251		χ^2^ = .200P = .655	H_1_ = .098, P = .754
**melatonin duration of secretion, hours**	13.18±.79	12.54±.38	11.08±.92	10.63±.79	H_1_ = 3.153, P = .076		χ^2^ = .200P = .655	H_1_ = .098,P = .754
**time of fitted melatonin maximum, clock time**	2.61±.69	3.65±.33	3.19±1.07	2.47±1.03	H_1_ = .844, P = .347		χ^2^ = .200P = .655	H_1_ = .884,P = .347
**melatonin amplitude, pg/ml**	55.82±7.95	61.37±6.44	41.57±9.16	37.19±9.01 a	H_1_ = 1.844, P = .175		χ^2^ = .200P = .655	H_1_ = .884,P = .347
**melatonin AUC, 24-hour**	925.44±120.27	984.66±151.36	769.34±142.33	660.29±132.44 b	H_1_ = 1.844, P = .175		χ^2^ = .200P = .655	H_1_ = 4.811,P = .028

PMDD: premenstrual dysphoric disorder; FP: follicular phase; LP: luteal phase; DLMOn, dim light melatonin onset; DLMOff, dim light melatonin offset; AUC, area under the curve.

Values are mean ± SEM.

a indicates different than controls at the level of P = .05 when LP values compared.

b indicates different than PMDD FP at level of P = .03.

#### Values across circadian phases

**Figure 1:**
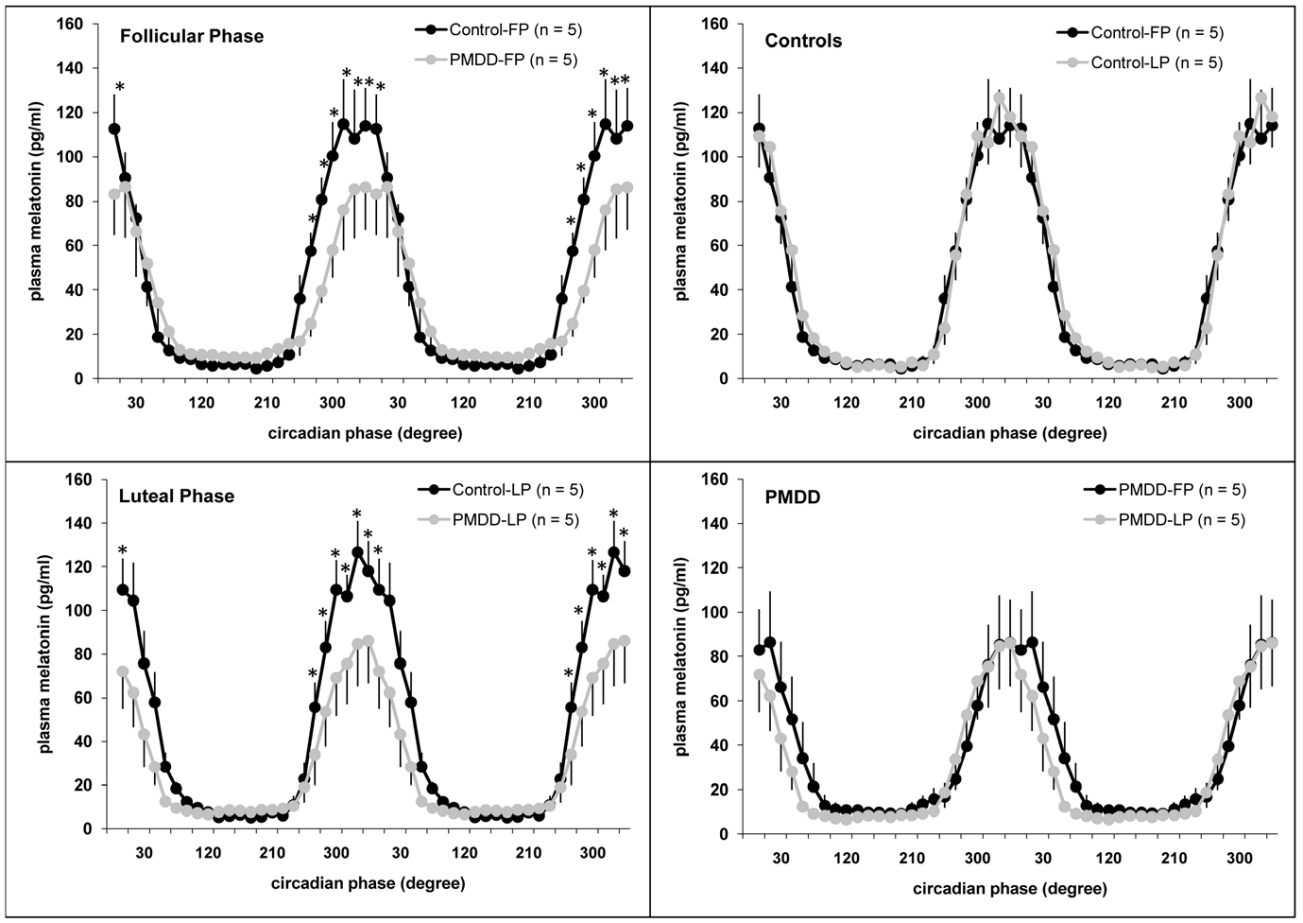
Circadian variation of plasma melatonin during the follicular and luteal phases in PMDD women and controls. * indicates significant differences between controls and PMDD women (P<.05). Data are double-plotted for illustration purposes. Values are mean ± SEM.

Three-way ANOVA revealed a significant group x circadian phase interaction (F_23,184_ = 2.24, P = .002) (Fig. 1), indicating a significant circadian variation (P<.001, simple main effects), with peak levels throughout the sleep period, in both groups, as well as significantly reduced melatonin levels in PMDD compared to controls at circadian phases centered in bins within the 270°−0° range (P<.05, simple main effects). A group x menstrual phase interaction (F_1,8_ = 5.11, P = .05) was also observed, with no menstrual phase variation of melatonin in controls (P = .31, simple main effects), but a trend for a menstrual phase variation of melatonin in PMDD (P = .07, simple main effects), with a reduction in LP vs. FP more apparent during the falling limb of the melatonin curve. A trend for a significant group x circadian phase x menstrual phase interaction (F_23,184_ = 1.55, P = .06) was observed for plasma melatonin. A significant main effect of circadian phase (F_23,184_ = 44.63, P<.01) was seen. No significant circadian phase x menstrual phase interaction (F_23,184_ = .68, P = .86), nor main effects of group (F_1,8_ = 1.83, P = .21) or menstrual phase (F_1,8_ = .51, P = .50), was observed.

#### Comparison of PMDD women and an expanded group of controls

**Figure 2:**
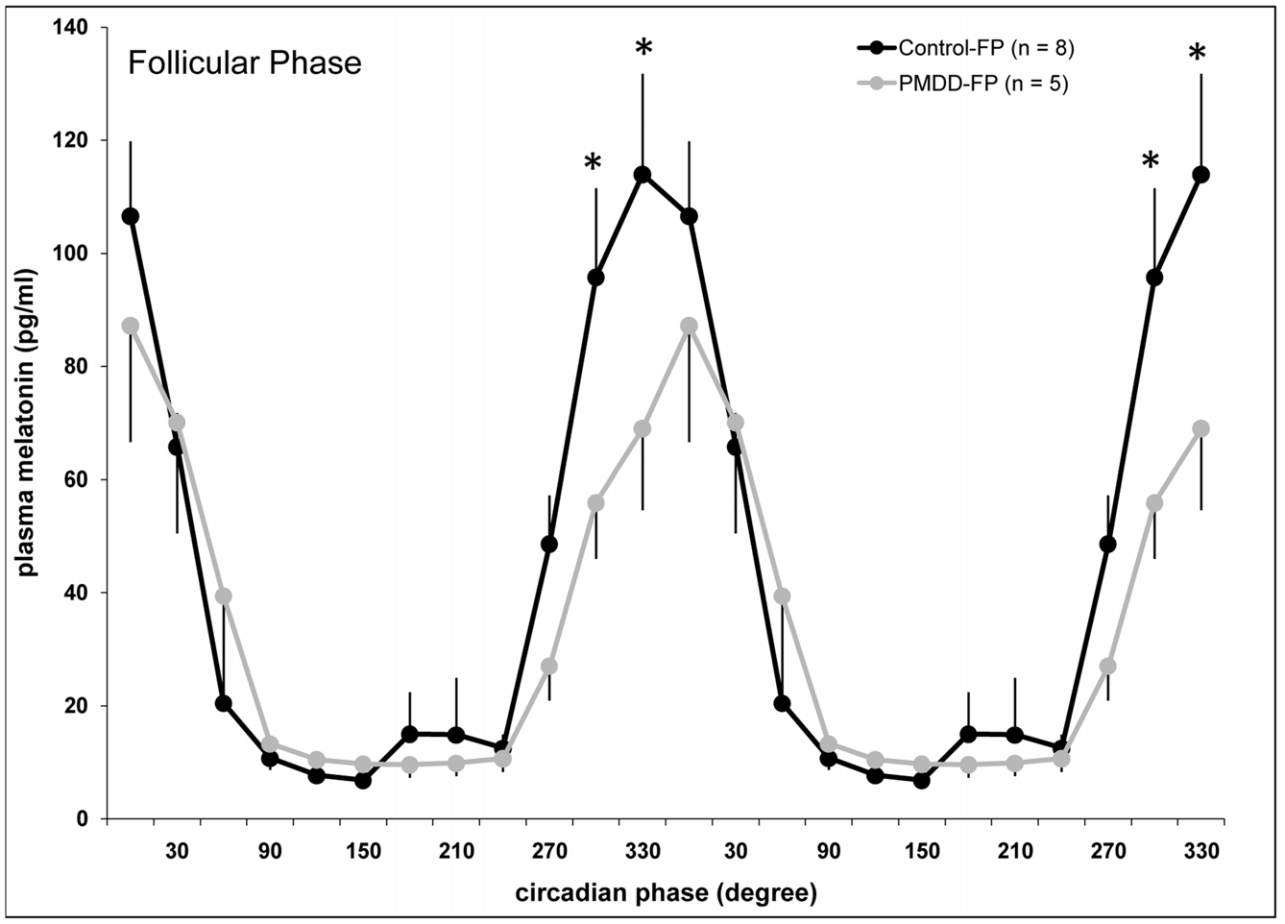
Circadian variation of plasma melatonin during the follicular phase in PMDD women and an expanded group of controls.^†^ * indicates significant differences between controls and PMDD women (P<.05). Data are double-plotted for illustration purposes. Values are mean ± SEM. ^†^ This figure presents FP data from the 5 control participants and 5 PMDD participants included in Figure 1, but the control group is supplemented by data from 3 other young healthy women who were studied under constant conditions in unrelated experiments. This larger group of 8 controls was compared to the 5 PMDD women of the current study during the FP in an attempt to increase sample size and further confirm results from our baseline group comparisons.

The baseline comparison of plasma melatonin during FP in PMDD and the expanded group of controls (n = 8) revealed a significant group x circadian phase interaction (F_11,121_ = 2.20, P = .02), indicating a significant circadian variation (P<.001, simple main effects) in both groups, as well as significantly reduced melatonin levels in PMDD compared to controls at circadian phases centered at 300° and 330° (P<.05, simple main effects) during FP (Fig. 2).

No significant differences were observed for DLMOn, DLMOff, duration of secretion, time of fitted maximum, amplitude, or AUC (Table 4).

**Table 4:** Circadian melatonin profile during the follicular phase in PMDD women and an expanded group of controls.†

	Controls, n = 8	PMDD women, n = 5	
	FP	FP	Controls vs. PMDD
**DLMOn, clock time**	20.82±.54	21.46±.66	t_11_ = −.74, P = .48
**DLMOff, clock time**	9.53±.44	8.29±.81	t_11_ = 1.47, P = .17
**melatonin duration of secretion, hours**	12.89±.51	11.08±.92	t_11_ = 1.88, P = .09
**time of fitted melatonin maximum, clock time**	3.07±.59	3.19±1.07	t_11_ = −.11, P = .92
**melatonin amplitude, pg/ml**	51.39±6.52	41.57±9.16	t_11_ = .90, P = .39
**melatonin AUC, 24-hour**	1142.46±193.82	769.34±142.33	t_11_ = 1.30, P = .22

PMDD: premenstrual dysphoric disorder; FP: follicular phase; DLMOn: dim light melatonin onset; DLMOff: dim light melatonin offset; AUC: area under the curve values are mean ± SEM.

† This table presents FP data from the 5 control participants and 5 PMDD participants included in Table 1, but the control group is supplemented by data from 3 other young healthy women who were studied under constant conditions in unrelated experiments. This larger group of 8 controls was compared to the 5 PMDD women of the current study during the FP in an attempt to increase sample size and further confirm results from our baseline group comparisons.

## Discussion

We quantified melatonin rhythms in a small group of PMDD women and compared it to that of controls at different menstrual phases, under highly controlled experimental conditions.

Our main finding was decreased melatonin in PMDD compared to controls throughout the nocturnal secretion period at both menstrual phases, and decreased amplitude of the circadian rhythm of plasma melatonin in PMDD compared to controls during LP. The endogenous melatonin rhythm is masked by postural changes [33] and light exposure [34], both of which were well-controlled for in this study. Reduced melatonin in PMDD vs. controls was initially reported [13], [14], but not in a recent study [35]. The reasons for this discrepancy are unclear, but the authors point out differences in radioimmunoassay kits, as well as methods of determining melatonin timing parameters that were assessed either quantitatively based on threshold values [13], [14] or qualitatively based on visual inspection [35]. Considering the small sample size of both groups in the present study, we cannot exclude the possibility that melatonin secretion might have been higher than usual in our controls. However, this is unlikely as the post-hoc analyses combining the data collected during the FP in the present study to those of three additional women studied either in a constant routine or CP protocol lead to similar conclusions.

Within the PMDD group, we found a menstrual variation for melatonin, with reduced AUC during LP vs. FP. Current results differ from others [13] who found no menstrual variation in PMDD, though not under constant conditions. Our results agree with a follow-up study which observed reduced AUC during LP compared to FP and reduced amplitude and mean levels during LP in women with PMDD [14]. In the aforementioned studies, as in ours, participants maintained their habitual sleep-wake patterns throughout the month-long study period. Before laboratory entry, our participants also underwent a rigorous preparatory phase, consisting of maintaining regular timing of sleep/dark and wake/light periods, with the aim of enforcing a stable entrainment of the endogenous circadian pacemaker to the sleep-wake schedule.

Reduced melatonin amplitude suggests either an impaired suprachiasmatic nucleus (SCN) signal that controls melatonin secretion, or a disturbance downstream of the SCN in the regulation of the overt melatonin rhythm in PMDD. Supporting a role of underlying clock dysfunction, Parry and colleagues [36] demonstrated a blunted and directionally altered melatonin phase-shift in response to morning bright light during LP in PMDD. Together, this decreased amplitude of pacemaker output and a compromised resetting capacity [36] may contribute to an increased susceptibility for mood disruption [3]. This interpretation is consistent with the report of higher prevalence of subsyndromal depression in low endogenous melatonin secretors compared to controls [37]. Reduced nocturnal melatonin secretion could contribute to alterations in sleep sometimes reported in PMDD [11].

PMDD shares similarities with MDD, in terms of mood symptomatology [38] and altered circadian physiology [12]. Like MDD, previous researchers have suggested a role of serotonergic dysregulation in the etiology of PMDD [39]. Decreases were observed for whole blood serotonin levels [40] and platelet serotonin uptake [41] in PMS compared to controls during the symptomatic phase. Furthermore, the efficacy of selective serotonin reuptake inhibitors (SSRIs) [42], fenfluramine [43], and L-tryptophan [22] in the treatment of PMDD symptoms also supports a central role of serotonin in PMDD.

Unfortunately, in the current study, we did not measure levels of serotonin, its metabolites, or markers of serotonergic activity. This precludes us from making any definitive statements on the role of serotonin in PMDD, and any proposed links between the serotonergic system and the melatonin system within PMDD patients is still speculative. Nevertheless, the serotonergic hypothesis is possibly supported by our findings of reduced melatonin secretion. Melatonin is synthesized in the pineal gland from the precursor serotonin [44], and is secreted in a circadian manner via inputs from the SCN. Melatonin seems to have a regulatory role on serotonin, as melatonin caused increased serotonin release from rat pineal glands in vitro [45], and pinealectomy in rats caused significant reductions in serotonin within the hypothalamus, midbrain and hippocampus [46]. Several anti-depressant drugs induce increased melatonin secretion, as illustrated by significant increases in plasma melatonin [47] and urinary 6-sulfatoxymelatonin [48] in depressed patients after treatment with a tricyclic antidepressant and MAO inhibitor, respectively. The characterization of agomelatine, an effective anti-depressive agent with MT_1_ and MT_2_ melatonin receptor agonist as well as 5-HT_2C_ receptor antagonist activity, also indicates a link between these two systems with ramifications for both circadian rhythms and mood disorders [49]. The presence of the 5-HT_2C_ receptor as well as MT_1_/MT_2_ receptors have been reported for various brain sites involved in circadian and mood regulation, including the SCN [50], [51], hippocampus [50], [52], nucleus accumbens [50], [52], and amygdala [50], [52]. Indeed, it is hypothesized that the combined, synergistic action of agomelatine on MT_1_/MT_2_ and 5-HT_2C_ receptors contributes to the clinical efficacy of the drug [49]. Considering the proposed relationships between melatonin and serotonin within the context of affective disorders, it would be interesting to determine whether SSRIs or agomelatine could be used as a chronotherapeutic to restore nocturnal levels of melatonin secretion. In SAD patients, fluoxetine appears to reduce melatonin [53], whereas fluvoxamine increases melatonin secretion, at least in healthy participants [54]. To our knowledge, the effect of agomelatine on endogenous melatonin secretion is still undetermined, though its effects, as well as those of different SSRIs, on melatonin secretion in PMDD should be pursued.

In the current study, nocturnal melatonin secretion in PMDD was reduced at both menstrual phases, compared to controls, which suggests a trait-vulnerability or a trait-marker for the disorder. Interestingly, the predisposition for an altered melatonin system in PMDD seems to be exacerbated by the onset of progesterone secretion during the symptomatic LP, when PMDD women showed a further reduction in circulating melatonin compared to their own FP. The presence of progesterone receptors at the SCN [55] may indicate a site of action, and a direct effect of progesterone on melatonin secretion is also possible since progesterone receptors were also localized at the bovine pineal gland [56]. Thus, fluctuations in sex hormones seem to alter the melatonin system, and may precipitate PMDD symptoms, in predisposed women. Ovulation during the experimental periods was confirmed in all participants via increased progesterone during LP compared to FP. Consistent with others [57], [58], we observed similar progesterone levels between controls and PMDD, which implies that a decrease in absolute circulating progesterone levels is unlikely to play a causative role in the development of PMDD symptoms. Nevertheless, we did observe here that whereas estradiol levels were non-significantly increased during LP compared to FP in controls, the menstrual phase difference in estradiol within PMDD women was much smaller. Throughout the course of a standard menstrual cycle, estradiol levels start to rise during the mid-FP and show a high peak a few days before ovulation. Estradiol levels then gradually decrease before reaching a secondary, lower level peak near the mid-LP [59]. In the current study, both groups were studied during their second CP visit around day 21 after menses onset (control range of days after menses: 19–25; PMDD range of days after menses: 19–23), placing them within the LP. Since estradiol was not sampled each day of the menstrual cycle, but only at two points, it is possible that PMDD women were studied during the rising or falling portion of the secondary LP peak time, thus accounting for their non-significantly lower levels compared to controls. Nonetheless, it is also possible that variations in estradiol levels are a characteristic or causative factor in the development of PMDD. Indeed, clinical efficacy has been demonstrated in the treatment of PMDD with a combination of drospirenone with ethinylestradiol, leading some authors to suggest that low estrogen levels may partially explain the mood symptoms observed in PMDD [60]. While the current study was not statistically powered or designed to make such a comparison, the possibility of a role of estradiol in the etiology of PMDD should be further studied in a larger group of women.

There were limitations in this study. While participants were studied in a controlled environment which limited the confounding effects of posture, activity, light, and meals, the inclusion of a nocturnal sleep episode may have influenced observed rhythms. Furthermore, given the small number of participants and the inter-individual variability in plasma melatonin levels, it is possible that, regardless of clinical diagnosis, the controls included in the study happened to be high melatonin secretors, or the limited number of PMDD women studied happened to be particularly low melatonin secretors. The first scenario is less likely, however, since, as described, FP melatonin levels were still higher in control vs. PMDD after the inclusion of data from three additional control women. It is also possible that, by chance, pre-study daytime light exposure in PMDD women was lower which could account for reduced nocturnal melatonin secretion in these women which is not pathophysiologically related to the disorder. Another limitation in the study related to small sample size in both groups is that the two-way and three-way ANOVAs used in some cases may not be ideal. Since there might not have been sufficient power to detect differences, there is also increased potential for a type II error. Nevertheless, a strength of the study is that all patients reached the criteria for PMDD diagnoses, as opposed to a more commonly utilized heterogeneous group comprising both PMS and PMDD. This patient-group homogeneity should help to limit variability in melatonin levels between PMDD women. We also utilized a within-subject design, whereby all participants were studied at both their follicular and luteal menstrual phases, which we believe adds to the statistical control of the study. Finally, this is, to our knowledge, the first investigation of plasma melatonin rhythms in PMDD under CP conditions, including time isolation. Despite the small sample size, the current report conducted under constant time isolation conditions is consistent with a larger study on melatonin secretion which utilized less vigorous circadian control [14].

Here, we described abnormal circadian melatonin secretion, which may relate to a serotonergic dysfunction in PMDD women. Findings of this pilot study indicate that pharmacological approaches such as exogenous nocturnal melatonin supplements, melatonin receptor agonists, or agomelatine should be further tested as therapeutic approaches for the management of depressive symptoms, and encourage more work on the chronobiological basis of psychiatric disorders including PMDD.
